# Stem cell factor and its soluble receptor (c-kit) in serum of asthmatic patients- correlation with disease severity

**DOI:** 10.1186/1471-2466-9-27

**Published:** 2009-06-01

**Authors:** Joanna S Makowska, Malgorzata Cieslak, Marek L Kowalski

**Affiliations:** 1 Department of Immunology, Rheumatology and Allergy, Medical University of Lodz, Lodz, Poland

## Abstract

**Background:**

SCF (stem cell factor) is a pleiotropic cytokine exerting its role at different stages of bone marrow development and affecting eosinophil activation, mast cells and basophil chemotaxis and survival. The aim of the study was to assess concentration of SCF and its soluble receptor c-kit (sc-kit) in peripheral blood of patients with asthma referring it to asthma severity and phenotype.

**Methods:**

The study involved 107 patients with bronchial asthma, well characterized with respect to severity and 21 healthy controls. Concentration of SCF and sc-kit in the patients serum were measured by ELISA method.

**Results:**

Mean serum SCF level in the group of asthmatics (n = 88) was significantly higher as compared to healthy controls (1010 pg/ml ± 37 vs 799 ± 33; p < 0,001). The level of SCF was higher in patients with severe asthma as compared to patients with non-severe asthma (1054 +/- 41 pg/ml vs 819 +/- 50; p < 0,01) and correlated with dose of inhaled glucocorticosteroids taken by the patients to achieve asthma control (R = 0,28; p < 0,01). The mean sc-kit serum level did not differ between asthmatic patients and healthy controls, however the level of sc-kit in non-severe asthmatics was significantly higher as compared to patients with severe asthma and healthy controls. In asthmatic patients (n = 63) the level of sc-kit correlated positively with FEV1% predicted value (R = 0,45; p < 0,001) and MEF25% predicted value (R = 0,33; p < 0,01). The level of sc-kit inversely correlated with the dose of inhaled glucocorticosteroids taken by the patients (R = -0,26; p < 0,01).

**Conclusion:**

Serum levels of SCF and its soluble receptor c-kit seem to be reflect asthma severity suggesting a role for these molecules in asthmatic inflammation.

## Background

Hemopoietic cytokines play a crucial role in the activation and survival of cells involved in asthmatic inflammation. Stem cell factor (SCF)- the c-kit ligand, although initially was described as a mast cell growth factor [1] appeared to be a pleiotropic cytokine exerting its role at the first stages of bone marrow stem cells development [2], inducing eosinophil activation [3] and basophil chemotaxis and survival [4].

As mast cells and eosinophils are key cells in the inflammatory process ongoing in the airways of patients with asthma the role of SCF in this disease has been studied. The expression of mRNA for SCF and its receptor c-kit were shown to be higher in the bronchi of patients with asthma as compared to controls [5] and serum SCF level was higher in patients with allergic asthma [6]. SCF is also overexpressed in nasal epithelia of patients with allergic rhinitis [7] and in the skin of patients with atopic dermatitis [8]. The expression of SCF is higher in nasal polyp epithelial cells of aspirin hypersensitive patients in comparison to aspirin tolerant patients and correlates with tissue eosinophils and polyp recurrences [9]. Studies on anti SCF therapy in mice model of asthma demonstrated that inhibition of SCF led to reduction of eosinophil accumulation in the airways, attenuation of peribronchial remodeling and decrease in bronchial hyperreactivity [10-12]. SCF is a cytokine exerting its role through binding to c-kit receptor [13], which can be shed from the cell surface and is detected in the serum as soluble c-kit (sc-kit) [14].

As SCF is the cytokine acting mainly in the bone marrow, we argue if the airway inflammation typical for asthma may lead to increase of the level of circulating SCF. So the aim of the study was to assess if the concentration of SCF and its soluble receptor c-kit in peripheral blood is increased in patients with asthma and if it correlates with disease severity and asthma phenotype.

## Methods

### Patients

The study involved 107 patients with asthma and 21 controls. SCF was assessed in 88 patients with asthma: 56 patients with severe and 32 patients with non-severe asthma, and the soluble c-kit level was assessed in 63 asthmatics: 41 severe and 22 non-severe (Table 1).

**Table 1:** Characteristics of severe and non-severe asthmatics in two subpopulations of patients in whom SCF and c-kit serum levels were determined (* p < 0,05)

	**SCF group (n = 88)**		**Sc-kit group (n = 63)**	
	
	**Non-severe asthma**	**Severe asthma**	**Non-severe asthma**	**Severe asthma**
**Number of patients**	32	56	22	41
**Female/male**	15/17	41/15	14/8	30/11
**Age mean (range)**	39,9 (19–57)	51 (23–74)	41 (24–64)	49,3 (22–74)
**Patients with positive SPT**	28 (87%)	39 (69%)	17	27
**Aspirin hypersensitivity**	15 (47%)	27 (48%)	5	18
**Nasal polyps**	13 (40%)	18 (32%)	4	10
**FEV1% of predictive value (mean)**	97%	73%*	98%	75%
**Patients on inhaled GCS (n)**	32	56	22	41
**-mean daily dose** **(mcg of budesonide)**	612	1990*	540	2035*
**Patients on oral GCS**	0	33*	0	22*
**-mean daily dose** **(mg of prednisone)**	0	7,79*	0	3,8*
**Total IgE (kU/ml)**	322,4 +/- 87	317,9 +/- 62	302,3+/- 57	335,9 +/- 92
**ECP (μg/ml)**	8,4 +/- 1,76	12,92 +/- 1,45	10,16 +/- 1,26	12,21 +/- 1,97
**Eosinophil count (cells/μl)**	266 +/- 62	351,4 +/- 36,5	290,8 +/- 46,5	313,8 +/- 40,25

The control group for SCF consisted of 21 healthy subjects (12 males and 9 females, mean age 35, range 21 to 46) and the control group for c-kit consisted of 15 healthy subjects (9 males and 6 females; mean age 34, range 21 to 45 years old) recruited from the healthy Lodz inhabitants. Both SCF and sc-kit were assessed in 44 patients and in 15 control subjects.

Asthma was diagnosed based on history, clinical examination and spirometric evaluation according to GINA definition. Severe refractory asthma was diagnosed according to ATS Workshop criteria which included use of oral glucocorticosteroids, high dose of inhaled glucocorticosteroids, daily use of a controller drug or short-acting beta 2 agonists, low respiratory function and history of exacerbation [15]. In all patients asthma was controlled or nearly controlled (group of severe asthmatics). Aspirin hypersensitivity was diagnosed based on convincing history of adverse reaction after use of nonsteroidal anti-inflammatory drugs and in patients with unequivocal history it was confirmed by bronchial challenge with lysine-aspirin. In all patients skin prick tests with panel of 14 standard allergens were performed. Oral and inhaled glucocorticosteroids (GCS) were withdrawn 12 hours, antileukotrienes 3 days and long acting beta 2 agonists 24 hours before blood collection. Research have been carried out in compliance with Helsinki Declaration. A study obtained approval of ethical committee of Medical University of Lodz. Every patient was informed about study protocol and procedures and signed informed consent form.

## Methods

Spirometry was carried according to ATS standards. Respiratory function (flow-volume curve) was measured with automatic spirometer (ABC Pneumo 2000RS, Poland) in patients who were without short acting beta 2 agonists for at least 8 h and long acting beta 2 agonists for at least 12 hours.

Blood (5 ml for serum) was collected by venipuncture, serum was separated and frozen in -20°C. The concentrations of SCF and sc-kit SCF in serum were measured by ELISA method (R&D systems; Minneapolis, USA).

The level of ECP and total IgE were measured by immunoenzymatic method (Pharmacia, Uppsala, Sweden). Total IgE and Eosinophil Cationic Protein(ECP) were measured in serum with ImmunoCAP (*Pharmacia Diagnostic, Sweden)*. Blood eosinophila (as a differential count) was assessed after staining with Pappenheim's method.

### Statistical analysis

Data are presented as mean ± standard error mean. For comparison of 3 groups of variables Kruskal Wallis ANNOVA and post hoc Dunn tests were used. For comparison of 2 variables nonparametric unpaired 2-group test (Mann- Whitney U test) was carried out. P < 0,05 was considered statistically significant. Pearson correlation coefficient was used to compare level of SCF and sc-kit with FEV1% of predicted value, MEF% of predicted value and dose of inhaled glucocorticosteroids received by the patients.

## Results

### SCF serum levels

**Figure 1:**
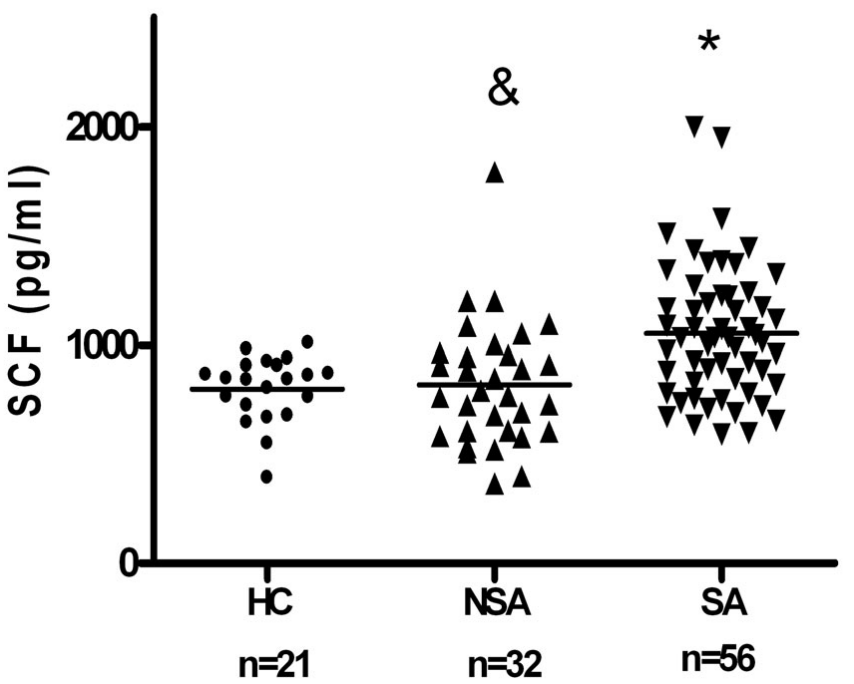
Serum SCF concentrations in patients with severe asthma (SA, n = 56) (* p < 0,001) and non-severe asthmatics (NSA, n = 32) (& p < 0,01) as compared to healthy controls (HC, n = 21).

Mean serum SCF level in asthmatics (n = 88) was significantly higher as compared to healthy controls (1010 +/- 37 pg/ml versus 799 +/- 33 pg/ml; p < 0,001). The level of SCF was higher in the group of patients with severe asthma as compared to patients with non-severe asthma (1054 +/- 41 pg/ml vs 819 +/- 50 pg/ml; p < 0,01) (Fig 1).

**Figure 2:**
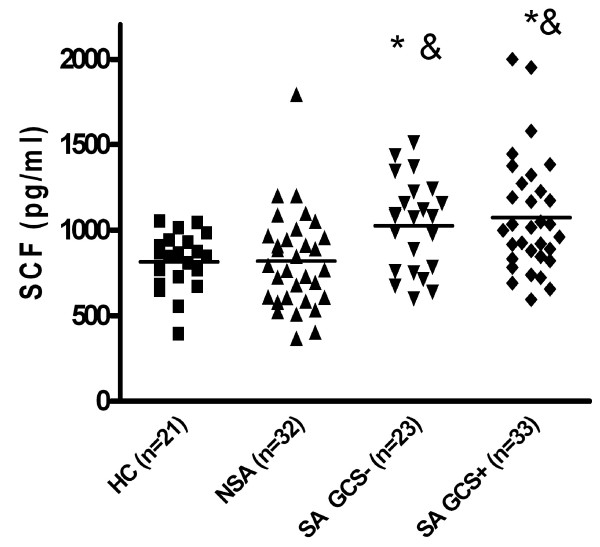
Serum SCF level in patients with severe asthma treated (SA GCS+) and not treated (SA GSC-) with oral glucocorticosteroids as compared to mild asthmatics (MA) (* p < 0,01) and nonatopic healthy controls (HC) (& p < 0,01).

When two subgroups of severe asthmatics: patients on oral gluccocorticosteroids (SA GCS+, n = 30) and patients not treated with oral gluccocorticosteroids (SA GCS-, n = 26) were analyzed, the SCF level was significantly higher in both groups (1066 +/- 66,41 pg/ml and 1023 +/- 45,9 pg/ml) as compared to mild asthmatics (819,3 +/- 50,3; p < 0,01) and healthy controls (815 +/- 36; p < 0,01) (Fig 2). In the group of severe asthma there was no differences in SCF level between patients on oral GCS (n = 33) and without oral GCS (n = 23) (1066 +/- 66,41 pg/ml versus 1023 +/- 45,9 pg/ml; ns).

**Figure 3:**
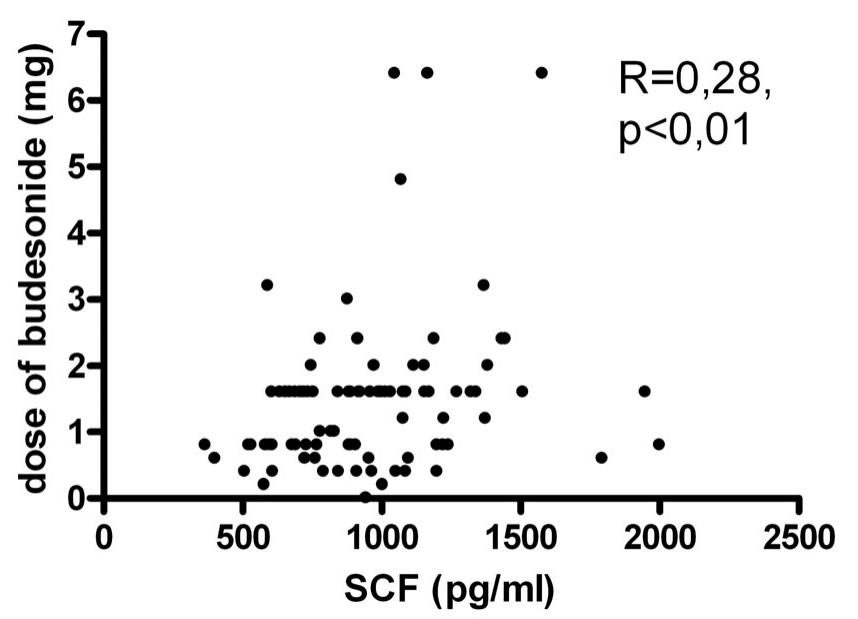
**Correlation between SCF serum concentrations and dose of inhaled steroids taken by the patients (n = 88)**.

For the whole group of asthmatic patients (n = 88) SCF level correlated with the dose of inhaled GCS taken by the patients to achieve asthma control (R = 0,28; p < 0,01) (Fig 3). SCF level did not correlate with FEV1% of predicted value, the number of eosinophils in peripheral blood or with ECP level in the serum. The SCF level did not differ between patients with or without aspirin hypersensitivity, with or without nasal polyps and patients with atopic and nonatopic asthma (table 2).

**Table 2:** The results of SCF and c-kit concentrations in the supgroups og aspirin hypersensitive and tolerant patients, patients with and without nasal polyps and atopic and nonatopic asthmatics.

**SCF (pg/ml)**			**c-kit (ng/ml)**		
**Aspirin hypersensitive (n = 42)**	**Aspirin tolerant (n = 46)**	**P value**	**Aspirin hypersensitive (n = 23)**	**Aspirin tolerant (n = 40)**	**P value**
959,1 +/- 44	980 +/- 50	Ns	76,9 +/- 6,8	71,7 +/- 5,7	ns

**With nasal polyps (n = 33)**	**Without nasal polyps (n = 55)**	**P value**	**With nasal polyps (n = 14)**	**Without nasal polyps (n = 49)**	**P value**
928 +/- 42,4	992 +/- 47,8	Ns	83,5 +/- 80	70,3 +/- 51,6	0,049

**Atopic (n = 67)**	**Nonatopic (n = 21)**	**P value**	**Atopic (n = 44)**	**Nonatopic (n = 19)**	**P value**
949,9 +/- 38	1033 +/- 65	ns	67,5 +/- 5,03	87 +/- 8,02	0,028

### Soluble c-kit serum level

**Figure 4:**
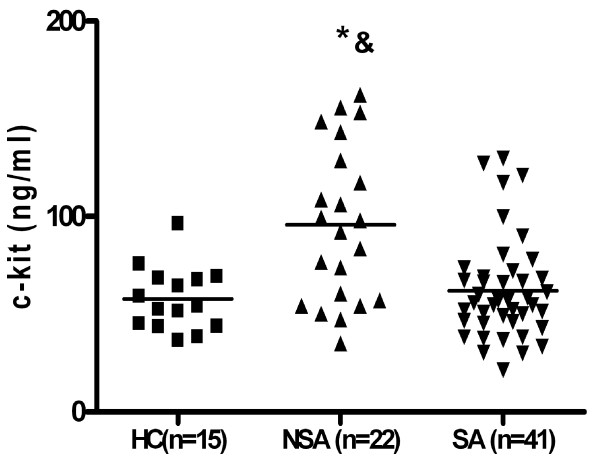
Soluble c-kit in serum non-severe asthmatics (NSA, n = 22) as compared to patients with severe asthma (SA, n = 41) (*p <0,05) and healthy controls (HC, n = 15) (& p < 0,01).

**Figure 5:**
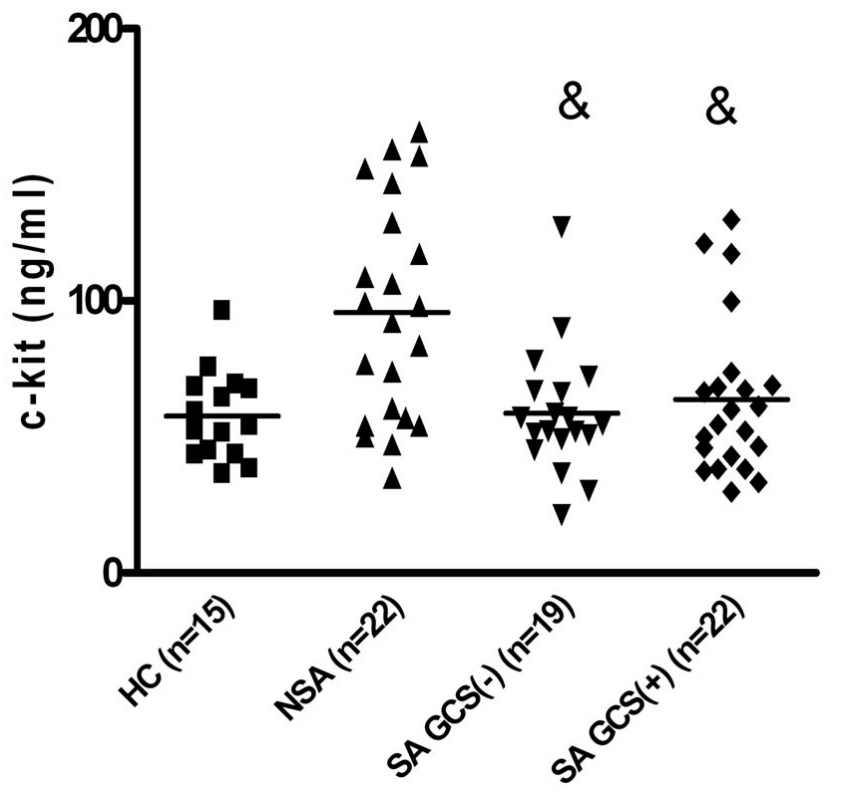
**Serum sc-kit level in patients with severe asthma treated (SA GCS+) and not treated (SA GSC-) with oral glucocorticosteroids as compared to mild asthmatics (MA) (& p < 0,01)**. Sc-kit level in mild asthmatics as compared to healthy control subjects (* p < 0,01).

The mean serum level of sc-kit in asthmatics without regard to asthma severity (n = 63) was similar to healthy controls (p > 0,05). In patients with non-severe asthma the mean sc-kit level (95,78 +/- 8,5 ng/ml) was higher as compared to patients with severe asthma (62,0 +/- 4,0 ng/ml; p < 0,01) and to healthy controls (57,7 +/- 4,18 ng/ml; p < 0,05). There was no difference between mean sc-kit level in patients with severe asthma and healthy controls (Fig 4). When two subgroups of severe asthmatics: patients on oral gluccocorticosteroids (SA GCS+, n = 21) and patients not treated with oral gluccocorticosteroids (SA GCS-, n = 19) were analyzed, the sc-kit level was significantly lower in both groups (64, +/- 5,9 9 ng/ml and 58,71 +/- 5,3 ng/ml) as compared to mild asthmatics (95,8 +/- 8,5; p < 0,01) (Fig 5). There was no differences in sc-kit level between patients on oral GCS and without oral GCS (mean 64, +/- 5,9 9 ng/ml versus 58,71 +/- 5,3 ng/ml; ns).

**Figure 6:**
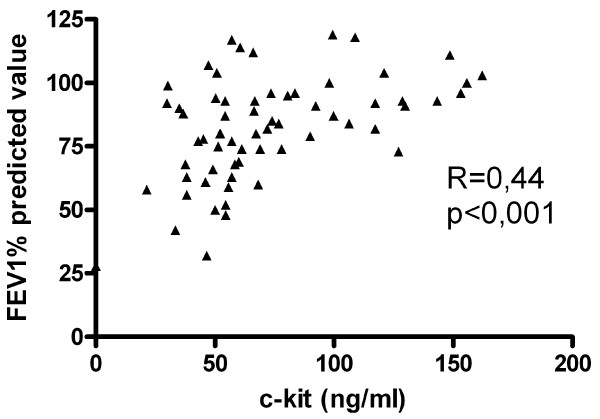
**Correlation of FEV1 % of predicted value with sc-kit serum level in asthmatic patients (n = 63)**.

**Figure 7:**
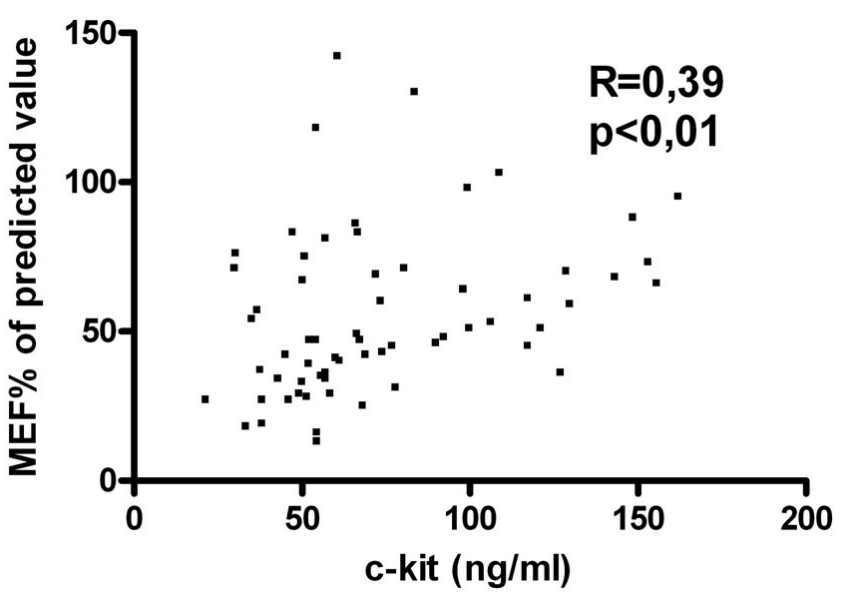
**Correlation of MEF25% with serum soluble c-kit in patients with asthma (n = 63)**.

**Figure 8:**
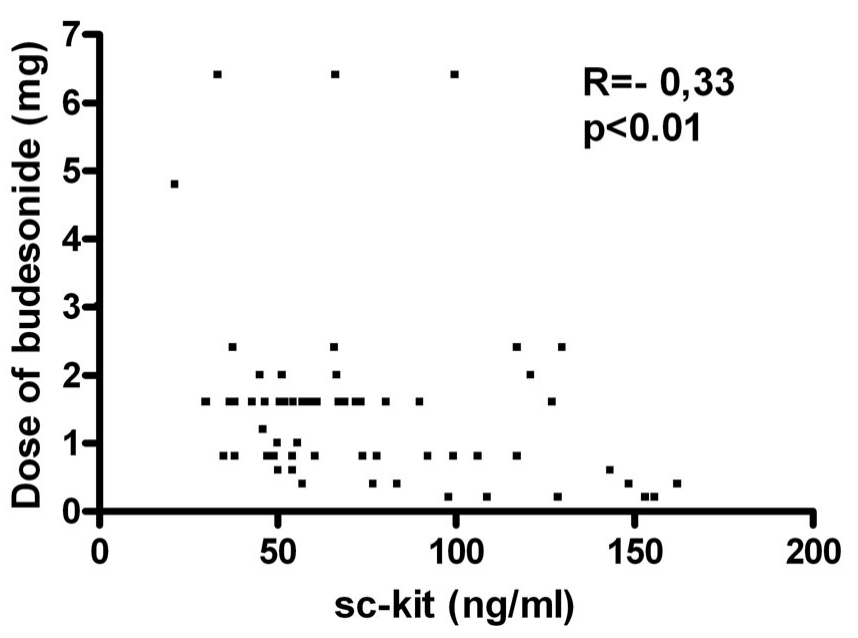
**Correlation of the dose of inhaled budesonide with soluble c-kit serum levels in patients with asthma (n = 63)**.

In asthmatic patients (n = 63) the level of sc-kit correlated positively with FEV1% of predicted value (R = 0,45; p < 0,001) (Fig 6) and with MEF25% of predicted value (R = 0,33; p < 0,01) (Fig 7), but correlated inversely with the dose of inhaled GCS taken by the patients (R = -0,26; p < 0,01) (Fig 8).

**Figure 9:**
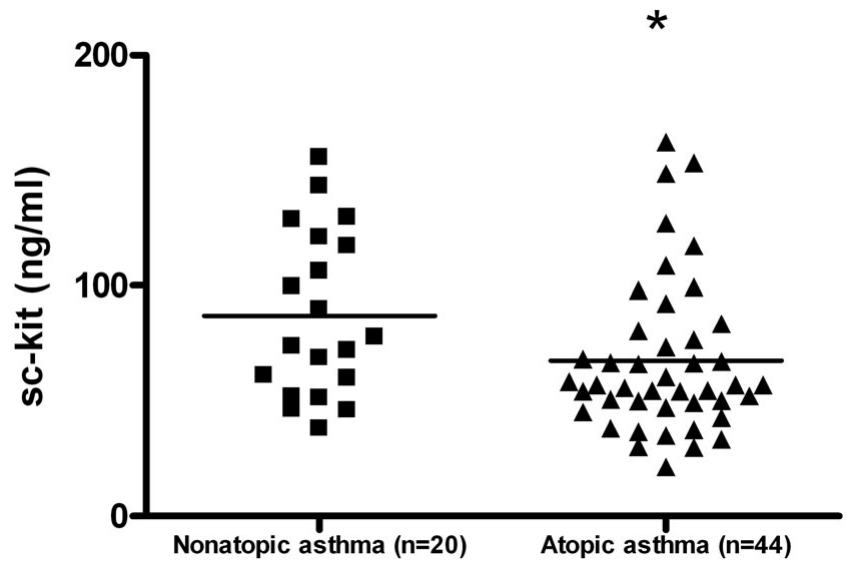
**Soluble c-kit in serum of patients with atopic and nonatopic asthma**.

**Figure 10:**
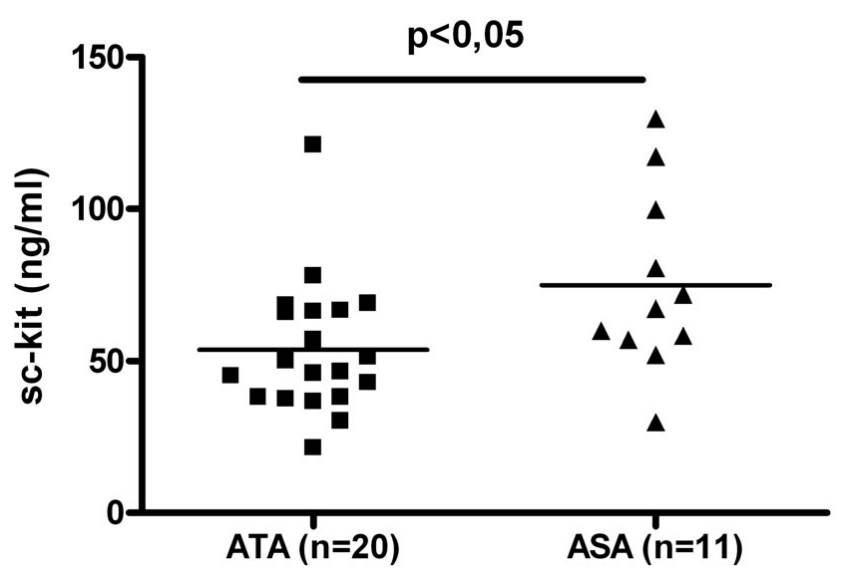
Soluble c-kit in serum levels in patients with asthma, aspirin hypersensitivity and nasal polyps (ASA group, n = 11) and in patients with asthma without aspirin hypersensitivity and nasal polyps (ATA group, n = 20) (* p < 0,05).

Sc-kit level was significantly higher in patients with nonatopic asthma as compared to patients with atopic asthma (87,00 +/- 8,02 ng/ml versus 67,50 +/- 5,03 ng/ml; p < 0,05) (Fig 9). Sc-kit level did not differ between the group of patients with or without aspirin hypersensitivity (table 2), however sc-kit level was significantly higher in patients with aspirin triad (n = 11) (74,3 ng/ml +/- 9 versus 53,8 +/- 4,9 ng/ml; p < 0,05) (Fig 10) and was significantly higher in patients with nasal polyps (table 2).

There was no correlation between serum SCF and sc-kit levels in patients with asthma, control subjects and in combined group (n = 59; r = -0,04, ns).

## Discussion

In this study we showed that serum level of SCF is higher in patients with asthma as compared to healthy subjects and is further increased in patients with severe refractory asthma. In parallel patients with severe asthma had decreased level of serum soluble c-kit as compared to non-severe asthmatics. Despite differences between SCF levels in patients with severe and non severe asthma serum SCF did not correlate with markers of asthma severity such as FEV1% and MEF% of predicted value, however it correlated with dose of inhaled corticosteroids needed to achieve asthma control.

Previous studies showed increased production of SCF in different allergic disease like asthma, allergic rhinitis and atopic dermatitis [5-8]. Expression of SCF and it's receptor c-kit mRNA in airway epithelium of patients with asthma was increased in comparison to healthy control airways [5]. The studies on SCF mRNA expression in nasal mucosa are unequivocal, one study showed overexpression of SCF mRNA in the nasal mucosa of patients with allergic rhinitis [7,16] while the other did not find the differences in the expression of SCF mRNA between patients with and without allergic rhinitis [17].

Although the serum level of SCF in healthy controls and patients with non- severe asthma was similar, the most severe patients had significantly higher serum SCF concentration in comparison both to healthy control and non-severe asthmatics. It is noticeable that despite aggressive anti-inflammatory treatment in the group of severe asthmatics (high doses of inhaled steroids, oral GCS, antileukotriens) the level of SCF was significantly increased. It has been demonstrated that at the beginning of the treatment GCS can increase the level of SCF [18], however they suppress the constitutive production of SCF [19]. Furthermore expression of SCF in asthmatic bronchi which is higher in comparison to healthy controls, can be normalized by treatment with inhaled glucocorticosteroids [20]. In contrast we did not see any difference in SCF serum levels between asthmatics with and without oral GCS.

As in asthmatic subjects not only SCF but also its receptor c-kit can be overexpressed on inflammatory cells we measured the soluble c-kit in the serum of patients [5]. Although the level of sc-kit was higher in the group of non-severe asthmatics as compared to healthy controls, in severe asthmatics sc-kit level was similar to healthy controls. Moreover, for the whole asthmatic group, the level of sc-kit positively correlated with such markers of disease severity as FEV1%, MEF25% of predicted value or the dose of inhaled glucocorticosteroids needed to maintain asthma control. It suggests that in non-severe asthmatics sc-kit could have protective role, e.g. by blocking SCF and therefore suppressing development of more severe inflammatory process. On the other hand the lack of increased sc-kit levels in patients with severe asthma could reflect decreased capacity of their serum to neutralize activity of SCF. Suggestion that, treatment with systemic glucocorticosteroids or high doses of inhaled glucocorticosteroids could be responsible for decreased generation of sc-kit is not likely since sc-kit levels were comparable in the group of patients treated and non-treated with oral GCS. Inhaled steroids seemed to suppress serum sc-kit levels in asthmatic patients. It suggests that inhaled and oral steroids may have different effect on serum sc-kit levels since patients treated and non-treated with oral GCS had similar sc-kit levels.

Our observations in line with study in patients with atopic dermatitis [8] which showed higher serum levels of both SCF and sc-kit as compared to control subjects and positive correlation of sc-kit levels with atopic dermatitis severity index SCORAD. On the other hand s-kit was decreased in patients with systemic lupus erythematosus and negatively correlated with disease severity [21].

We were not able to correlate the level of SCF with aspirin hypersensitivity or concomitant nasal polyposis, although in previous study the increased expression of SCF within the nasal polyps of patients with asthma and aspirin hypersensitivity was reported [9]. It suggests that serum levels of SCF may not fully reflect local production of SCF in the inflammatory tissue. In contrast to previous study SCF levels in peripheral blood were significantly higher than in healthy controls only in the group of patients with allergic asthma, SCF levels did not differ significantly between patients with allergic and nonallergic asthma [6] which was also observed in our study.

## Conclusion

Our study demonstrated that serum levels of SCF, a pleiotropic cytokine influencing development of two major cell lines in asthmatic inflammation- mast cells and eosinophils is elevated in patients with most severe asthma and may be important in pathogenesis of airway obstruction. Furthermore it seems that physiological down regulation of this cytokine by soluble kit receptor is also impaired in patients with severe asthma, thus modifying the SCF/c-kit interaction in asthmatics subjects.

## Competing interests

The authors declare that they have no competing interests.

## Authors' contributions

All authors read and approved the final manuscript.

## Pre-publication history

The pre-publication history for this paper can be accessed here:



## References

[B1] Galli SJ, Zsebo KM, Geissler EN (1994). The kit ligand, stem cell factor. Adv Immunol.

[B2] Möhle R, L K (2007). Hematopoietic growth factors for hematopoietic stem cell mobilization and expansion. Semin Hematol.

[B3] Oliveira SH, Taub DD, Nagel J, Smith R, Hogaboam CM, Berlin A, Lukacs NW (2002). Stem cell factor induces eosinophil activation and degranulation: mediator release and gene array analysis. Blood.

[B4] Heinemann A, Sturm GJ, Ofner M, Sturm EM, Weller C, Peskar BA, Hartnell A (2005). Stem cell factor stimulates the chemotaxis, integrin upregulation, and survival of human basophils. J Allergy Clin Immunol.

[B5] Al-Muhsen SZ, Shablovsky G, Olivenstein R, Mazer B, Q H (2004). The expression of stem cell factor and c-kit receptor in human asthmatic airways. Clin Exp Allergy.

[B6] Lei Z, Liu G, Huang Q, Lv M, Zu R, Zhang GM, Feng ZH, Huang B (2008). SCF and IL-31 rather than IL-17 and BAFF are potentialindicators in patients with allergic asthma. Allergy.

[B7] Kim YK, Nakagawa N, Nakano K, Sulakvelidze I, Dolovich J, Denburg J (1997). Stem cell factor in nasal polyposis and allergic rhinitis: increased expression by structural cells is suppressed by in vivo topical corticosteroids. J Allergy Clin Immunol.

[B8] Kanabe T, Soma Y, Kawa Y, Kashmida M, Mizoguchi M (2001). Serum levels of soluble stem cell factor and soluble KIT are elevated in patients with atopic dermatitis and correlate with the disease severity. Br J Dermatology.

[B9] Kowalski ML, Lewandowska-Polak A, Woźniak J, Ptasiñska A, Jankowski A, Wagrowska-Danilewicz M, Danilewicz M, Pawliczak R (2005). Association of stem cell factor expression in nasal polyp epithelial cells with aspirin sensitivity and asthma. Allergy.

[B10] Berlin AA, Hogaboam CM, Luckas NW (2006). Inhibition of SCF attenuates peribronchial remodeling in chronic cockroach allergen-induced asthma. Laboratory Investigation.

[B11] Berlin AA, Lincoln P, Tomkinson A, Lukacs NW (2004). Inhibitionof stem cell factor reduces pulmonary cytokines levels during allergic airway responses. Clin Exp Immunol.

[B12] Berlin AA, Lukacs NW (2005). Treatment of cockroach allergen asthma model with imatinib attenuates airway responses. Am J Resp Crit Care Med.

[B13] Langley KE, Bennett LG, Wypych J, Yancik SA, Liu XD, Westcott KR, Chang DG, Smith KA, Zsebo KM (1993). Soluble stem cell factor in human serum. Blood.

[B14] Wypych J, Bennett LG, Schwartz MG, Clogston CL, Lu HS, Broudy VC, Bartley TD, Parker VP, Langley KE (1995). Soluble Kit receptor in human serum. Blood.

[B15] Wenzel SE, Fahy JV, Irvin C, Peters SP, Spector S, Szefler SJ (2000). Proceedings of the ATS Workshop on Refractory Asthma. Current Understanding, Recommendations, and Unanswered Questions. Am J Resp Crit Care Med.

[B16] Otsuka H, Kusumi T, Kanai S, Koyama M, Kuno Y, Takizawa R (1998). Stem cell factor mRNA expression and production in human nasal epithelial cells: contribution to the accumulation of mast cells in the nasal epithelium of allergy. J Allergy Clin Immunol.

[B17] Salib RJ, Kumar S, Wilson SJ, Howarth PH (2004). Nasal mucosal immunoexpression of the mast cell chemoattractants TGF-beta, eotaxin, and stem cell factor and theire receptors in allergic rhinitis. J Allergy Clin Immunol.

[B18] Da Silva CA, Kassel O, Lebouquin R, Lacroix EJ, Frossard N (2004). Paradoxical early glucocorticosteroid induction of stem cell factor (SCF)expression in inflammatory conditions. Br J Pharmacol.

[B19] Kassel O, Schmildlin F, Duvernelle C, de Blay F, Frossard N (1998). Up- and down-regulation of glucocorticosteroids of the constitutive expression of the mast cell growth factor stem cell factor by human lung fibroblasts in culture. Mol Pharmacol.

[B20] Da Silva CA, Blay F, Izrael-Biet D, Laval AM, Glasser N, Pauli G, Frossard N (2006). Effect of glucocorticosteroids on stem cell factor expression in human asthmatic bronchi. Clin Exp Allergy.

[B21] Kitoh T, Ishikawa H, Sawada S, Koshino K, Tokano Y, Hashimoto H, Nakagawa S (1998). Significance of stem cell factor and soluble KIT in patients with systemic lupus erythematosus. Clin Rheumatol.

